# Modulation of Apoptotic, Cell Cycle, DNA Repair, and Senescence Pathways by Marine Algae Peptides in Cancer Therapy

**DOI:** 10.3390/md22080338

**Published:** 2024-07-25

**Authors:** Visuddho Visuddho, Princella Halim, Helen Helen, Adi Muradi Muhar, Muhammad Iqhrammullah, Nelly Mayulu, Reggie Surya, Raymond Rubianto Tjandrawinata, Rosy Iara Maciel Azambuja Ribeiro, Trina Ekawati Tallei, Nurpudji Astuti Taslim, Bonglee Kim, Rony Abdi Syahputra, Fahrul Nurkolis

**Affiliations:** 1 Faculty of Medicine, Universitas Airlangga, Surabaya 60132, Indonesia; 2 Department of Pharmacology, Faculty of Pharmacy, Universitas Sumatera Utara, Medan 20155, Indonesia; 3 Faculty of Medicine, Universitas Sumatera Utara, Medan 20155, Indonesia; 4 Postgraduate Program of Public Health, Universitas Muhammadiyah Aceh, Banda Aceh 23123, Indonesia; 5 Department of Nutrition, Faculty of Health Science, Muhammadiyah Manado University, Manado 95249, Indonesia; 6 Department of Food Technology, Faculty of Engineering, Bina Nusantara University, Jakarta 11480, Indonesia; 7 Department of Biotechnology, Faculty of Biotechnology, Atma Jaya Catholic University of Indonesia, Jakarta 12930, Indonesia; 8 Experimental Pathology Laboratory, Midwest Campus, Federal University of São João del-Rei, Divinópolis 36301-158, Brazil; 9 Department of Biology, Faculty of Mathematics and Natural Sciences, Sam Ratulangi University, Manado 95115, Indonesia; 10 Division of Clinical Nutrition, Department of Nutrition, Faculty of Medicine, Hasanuddin University, Makassar 90245, Indonesia; 11 Department of Pathology, College of Korean Medicine, Kyung Hee University, Seoul 02447, Republic of Korea; 12 Department of Biological Sciences, Faculty of Sciences and Technology, State Islamic University of Sunan Kalijaga (UIN Sunan Kalijaga), Yogyakarta 55281, Indonesia; fahrul.nurkolis.mail@gmail.com

**Keywords:** marine algae, peptides, anticancer, apoptosis, nanocarrier formulation, drugs discovery

## Abstract

Marine algae, encompassing both macroalgae and microalgae, have emerged as a promising and prolific source of bioactive compounds with potent anticancer properties. Despite their significant therapeutic potential, the clinical application of these peptides is hindered by challenges such as poor bioavailability and susceptibility to enzymatic degradation. To overcome these limitations, innovative delivery systems, particularly nanocarriers, have been explored. Nanocarriers, including liposomes, nanoparticles, and micelles, have demonstrated remarkable efficacy in enhancing the stability, solubility, and bioavailability of marine algal peptides, ensuring controlled release and prolonged therapeutic effects. Marine algal peptides encapsulated in nanocarriers significantly enhance bioavailability, ensuring more efficient absorption and utilization in the body. Preclinical studies have shown promising results, indicating that nanocarrier-based delivery systems can significantly improve the pharmacokinetic profiles and therapeutic outcomes of marine algal peptides. This review delves into the diverse anticancer mechanisms of marine algal peptides, which include inducing apoptosis, disrupting cell cycle progression, and inhibiting angiogenesis. Further research focused on optimizing nanocarrier formulations, conducting comprehensive clinical trials, and continued exploration of marine algal peptides holds great promise for developing innovative, effective, and sustainable cancer therapies.

## 1. Introduction

Cancer is particularly a significant issue in the 21st century, affecting society, public health, and the economy. It is responsible for around 16.8% of all fatalities and 22.8% of deaths from noncommunicable diseases (NCDs) globally. The condition is also responsible for 30.3% of premature deaths from NCDs worldwide, affecting individuals between the ages of 30 and 69 years. In 117 countries (out of 183 countries), cancer is among the top three causes of death in the age group [1].

As of 2022, the latest worldwide data reveals that there were about 20 million newly diagnosed cancer cases and 9.7 million cancer-related deaths. Based on demographic projections, it is predicted that the yearly incidence of cancer would rise to 35 million by 2050, representing a 77% increase compared to the number of new cases in 2022. The global prevalence of cancer and the variation in cancer characteristics across different regions and levels of human development highlight the necessity for a worldwide increase in focused cancer control strategies. Investing in preventive measures, such as addressing significant risk factors for cancer like smoking, overweight and obesity, and infections, has the potential to avoid millions of future cancer cases and save numerous lives globally [2].

Chemotherapy is largely regarded as the most efficient and commonly utilized treatment for cancers, either as a standalone therapy or in conjunction with radiotherapy. Several chemotherapy drugs are utilized in the treatment of cancer, one of them is anthracycline [3]. Anthracyclines are a category of chemotherapy medications that are composed of antibiotics obtained from the Streptomyces bacterium. Doxorubicin, epirubicin, and idarubicin are all examples of anthracyclines. These substances are extremely efficient in treating many different types of malignancies by causing damage to the DNA strands through the creation of unstable oxygen molecules, which in turn interferes with the process of DNA replication. Anthracyclines exert their anticancer effect by intercalating between DNA base pairs and inhibiting DNA topoisomerase II, a crucial enzyme involved in DNA replication and transcription. However, the administration of anthracycline medicines is closely constrained due to their ability to also affect cardiac cells and cause cardiotoxicity, potentially resulting in heart failure [4]. Due to the toxicity and side effects induced by current chemotherapy drugs, it is still crucial to find other alternatives, particularly from natural products, including marine algal peptides.

Marine algae, one of the biotechnological explorations, is a promising and huge natural source for anticancer compounds. Global microalgae production is anticipated to hit 56,456 tons. China leads the top ten production with 54,850 tons, followed by the Central African Republic, Bulgaria, Greece, Tunisia, Burkina Faso, Central African Republic, and Spain. Recently, there has been a lot of interest in identifying medicinally valuable compounds, especially those with potential anticancer properties, because of the structural diversity and distinctiveness of these molecules [5,6].

There are two types of marine algae, macroalgae and microalgae. Both contain a wide variety of biomolecules, some of which have strong anticancer properties, including alkaloid, fatty acids, phenolics, terpenes, sulfated polysaccharides (SPs), carotenoids, sterols, and phycobiliproteins [5,7,8]. The utilization of marine algal in drug development presents several benefits, such as their rapid generation time, metabolic flexibility, lack of rivalry for arable land, ability to grow in any season, and minimal need for specialized nutrients [8]. Likewise, bioactive substances discovered in algae have been witnessed to possess anticancer capabilities by causing apoptosis and preventing cell division through disrupted signaling pathways [6].

**Figure 1:**
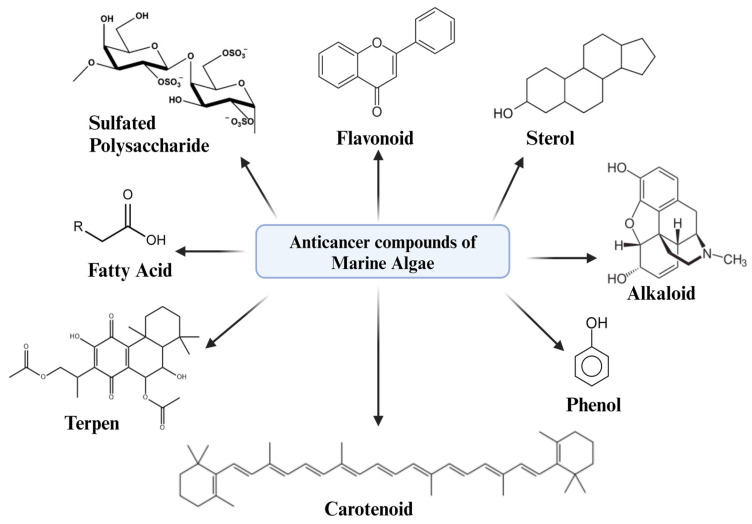
Several anticancer compound structures found in marine algae.

Furthermore, marine algal pharmaceutical compounds have shown potential in anti-inflammation and antioxidant properties. These compounds regulate reactive oxygen species, which influence carcinogenesis and cancer development. Marine algae extracts have shown promise in inhibiting malignant cell growth or promoting apoptosis in human cancer cell lines (Figure 1), with a specific focus on pro-oxidant natural products [9,10,11]. Developing marine algae as an effective and environmentally sustainable “bio factory” of bioactive compounds with antioxidant activity is a biotechnological challenge, given the smaller potential of a single microalgae cell in comparison to that of a multicellular plant [9]. On the other hand, in comparison with other bioactive sources, marine algae relatively have not received much attention in regard to anticancer drugs development (Figure 1).

This review specifically discusses anticancer compounds derived from marine algae. In this review, topics such as current developments in this area are presented and discussed along with experimental findings and specific peptides mechanism. Considering these considerations, bridging the gap between marine algae peptides and current anticancer drug discovery emerges as a critical imperative. Integrating insights from marine algae peptides bioactivity with contemporary research can offer novel avenues for developing more effective cancer treatments. Such an approach holds promise in addressing the limitations of current therapies, potentially revolutionizing cancer management and improving outcomes for affected individuals worldwide.

## 2. Marine Algal Peptides

**Figure 2:**
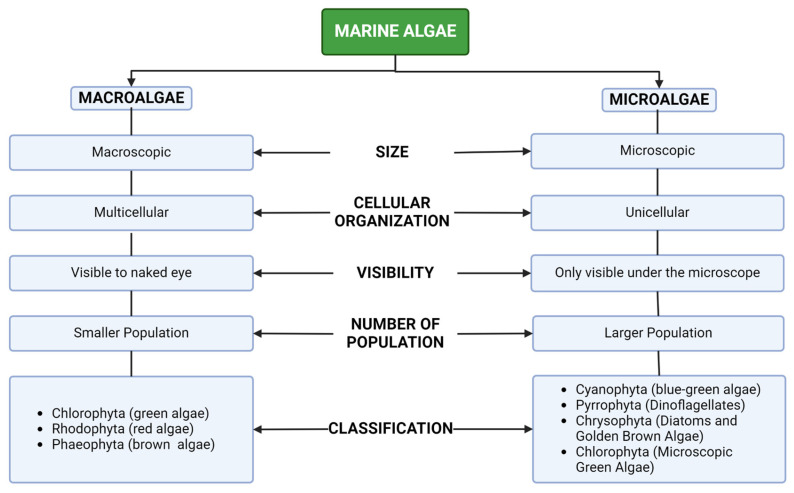
The difference between macroalgae and microalgae.

Algae are part of the plant kingdom’s earliest evolutionary tiers and are different in their ability to photosynthesize. Algae are divided into two categories: macroalgae and microalgae (Figure 2). Macroalgae, frequently referred to as “seaweeds” are multicellular marine creatures that resemble large plants. Color-based classifications include Rhodophyta, Chlorophyta, and Phaeophyta, also known as red, green, and brown algae, respectively [12]. Meanwhile, microalgae are small photosynthetic organisms that live in both saltwater as well as freshwater environments that belong to a varied group of organisms, including photoautotrophic protists such as prokaryotic cyanobacteria, which are additionally known as blue-green algae. The distinct features between macroalgae and microalgae are presented in Figure 2. Microalgae account for almost 70% of global biomass, and they generate molecules like carbohydrates, protein, and lipids. Microalgae are photosynthetic micro-organisms with a lack of cell organelles compared to land-based plants. Microalgae can grow via photosynthesis in the presence of CO_2_, solar light, and water. The cultivation can be carried out in marginal ponds, raceway ponds, and synthetic tanks [13].

Peptides are an important bioactive compound found in several marine organisms and have been extensively researched [14]. Bioactive peptides typically include 2–20 amino acid residues. Bioactive peptides can be released through three methods: solvent extraction, enzymatic hydrolysis, or microbial fermentation [15]. Marine algae are one of these organisms that are useful in pharmaceutical biotechnology and drug discovery [16]. Marine bioactive peptides are gaining popularity in pharmaceutical, cosmetic, and nutraceutical product development due to their unique biological features. They play crucial roles in the algae’s survival systems, such as defense, reproduction, growth, and homeostasis [17]. Algal species contain bioactive compounds that have been evidenced to have significant antidiabetic, antihypertensive, and antibacterial and antiviral properties, as well as neuroprotective effects [12,18]. For example, seaweed has been found to have bioactive peptides with antihypertensive, antioxidant, and antidiabetic properties [17]. The majority of peptides have anticancer activity by upregulating the apoptosis pathway and downregulating the proliferation pathway. Table 1 provides detailed information about the mechanism of action and IC_50._

**Table 1:** List of peptides identified in various algae species and their known bioactivities.

Bioactivity	Peptide Name or Sequence	Source	Enzymatic Treatment/Cell Lines	IC_50_	Mechanism of Action	References
Antiartherosclerosis	NIGK	*Palmaria palmata*	Papain	2.32 mM **	↓ PAG-AH	[13]
Antiartherosclerosis	VECYGPNRPQF	*Chlorella* sp.	Pepsin, Flavourzyme, Alcalase, and Papain	2.32 mM **	↓ VCAM (E-selectin, ICAM, VCAM, MCP-1 and ET-1) gene expression	[14]
Antiartherosclerosis	LDAVNR, MMLDF	*Spirulina maxima*	Trypsin, α-chymotrypsin, and pepsin	2.32 mM **	↓ IL-6, IL-8, MCP-1, P-selectin, ROS, and Egr-1	[15,16]
Anticancer	Isomalyngamide A and A-1	*Lyngbya majuscula*	MDA-MB-231	0.06—0.337 μM	↓ VEGFR2, MMP-9	[17]
Anticancer	Cocosamides A-B	*Lyngbya majuscula*	MCF7	A:30 μM; B:39 μM	↓ cell viability	[18]
Anticancer	VECYGPNRPQF	*Chlorella vulgaris*	Pepsin	70 μg/mL **	↑ antiproliferation, post-G1 cell cycle arrest	[19]
Anticancer	Desmethoxymajusculamide C	*Lyngbya majuscula*	MDA-MB-435	0.22 µM **	Actin microfilament disruption	[20]
Anticancer	Polypeptide CPAP	*Chlorella pyrenoidosa*	Papain, trypsin, and alcalase	426 μg/mL **	↑ apoptosis	[21]
Anticancer	Polypeptide Y2	*Spirulina platensis*	Trypsin, alcalase, pepsin, and papain	61 μg/mL **	↑ apoptosis	[22]
Antihypertensive	Gln-Val-Glu-Tyr	*Gracilariopsis lemaneiformis*	Trypsin, favourzyme, papain, alkaline protease	474.36 μM **	↑ ACE-I, ↓ BP	[23]
Antihypertensive	FGMPLDR MELVLR	*Ulva intestinalis*	Protein hydrolysates	219.35 μM **	↑ ACE-I, ↓ BP	[24]
Antihypertensive	Val-Glu-Gly-Tyr	*Chlorella ellipsoidea*	Alcalase	128.4 mM **	↓ radical formation, ROS	[25]
Antihypertensive	Ile-Pro	*Ulva rigida*	Bromelain, chymotrypsin, ficin, pancreatin, pepsin, peptidases, protease, trypsin	87.6 μM **	↑ ACE-I, ↓ BP	[26]
Antihypertensive	Ala-Phe-Leu	*Ulva rigida*	Bromelain, chymotrypsin, ficin, pancreatin, pepsin, peptidases, protease, trypsin	65.8 μM **	↑ ACE-I, ↓ BP	[26]
Antihypertensive	Gly-Met-Asn-Asn-Leu-Thr-Pro	*Nannochloropsis oculata*	Pepsin	123 mM **	↑ Bioavailbility, ↓ BP	[27]
Antihypertensive	Leu-Glu-Gln	*Nannochloropsis oculata*	Pepsin	173 mM **	↑ Bioavailbility, ↓ BP	[27]
Antihypertensive	Val-Glu-Cys-Tyr-Gly-Pro Asn-Arg-Pro-Gln-Phe	*Chlorella vulgaris*	Pepsin	29.6 mM **	↓ BP	[19]
Antihypertensive	Ile-Val-Val-Glu	*Chlorella vulgaris*	Pepsin	315.3 mM **	↑ ACE-I, ↓ BP	[28]
Antihypertensive	Ile-Ala-Glu	*Spirulina platensis*	Pepsin	34.7 mM **	↑ ACE-I, ↓ BP	[28]
Antihypertensive	Ala-Phe-Leu	*Chlorella vulgaris*	Pepsin	63.8 mM **	↑ ACE-I, ↓ BP	[28]
Antihypertensive	Phe-Ala-Leu	*Spirulina platensis*	Pepsin	11.4 mM **	↑ ACE-I, ↓ BP	[28]
Antihypertensive	Phe-Ala-Leu	*Chlorella vulgaris*	Pepsin	26.3 mM **	↑ ACE-I, ↓ BP	[28]
Antihypertensive	Ala-Glu-Leu	*Spirulina platensis*	Pepsin	11.4 mM **	↑ ACE-I, ↓ BP	[28]
Antihypertensive	Ala-Glu-Leu	*Chlorella vulgaris*	Pepsin	57.1 mM **	↑ ACE-I, ↓ BP	[29]
Antihypertensive	Ile-Ala-Pro-Gly	*Spirulina platensis*	Pepsin	11.4 mM **	↑ ACE-I, ↓ BP	[29]
Antihypertensive	Val-Val-Pro-Pro-Ala	*Chlorella vulgaris*	Pepsin	79.5 mM **	↑ ACE-I, ↓ BP	[29]
Antihypertensive	Val-Ala-Phe	*Spirulina platensis*	Pepsin	35.8 mM **	↑ ACE-I, ↓ BP	[29]
Antihypertensive	YH, KY, FY, IY	*Undaria pinnatifida*	No enzyme use	2.7–43.7 μmol/L	↑ ACE-I, ↓ BP	[30]
Antioxidant	Protease extract	*Scytosiphon lomentaria*	Multienzyme complex	<125 µg/mL **	↑ radical scavenging, ↑ antioxidative	[31]
Antioxidant	VECYGPNRPQF	*Chlorella vulgaris*	Pepsin	* ND	↓ superoxide radical quenching growth, ↓ cell cycle arrest	[32,33]
Antioxidant	Enzymatic digests	*Ishige okamurae*	Multienzyme complex	<25 µg/mL **	↑ antioxidative	[34]
Antioxidant	NIPP-1 (Pro-GlyTrp-Asn-Gln-Trp-Phe-Leu), and NIPP-2 (Val-Glu-Val-Leu-Pro-Pro-Ala-Glu-Leu)	*Naviculla incerta*	Papain	* ND	Cytotoxic	[35]
Antioxidant	Phe-Ser-Glu-Ser-Ser-Ala-Pro-Glu-Gln-His-Tyr	*Spirulina platensis*	Thermolysin	171.47 µg/mL **	↑ antioxidant	[36]
Immunomodulatory	Protein hydrolysates	*Ecklonia cava*	Kojizyme	* ND	↑ lymphocytes, monocytes, granulocytes; ↓ regulation of TNF-α, IFN-γ; ↑ regulation of IL-4, IL-10	[37]
Immunomodulatory	Protein hydrolysates	*Porphyra columbina*	trypsin, alcalase	2.1–5.6 g/L **	↓ TNF, IFN-γ; ↑IL-10	[38]
Immunomodulatory	Protein hydrolysates	*Chlorella vulgaris*	pancreatin	* ND	↑ humoral and cell-mediated immune functions (TDAR, DTHR)	[39]

Abbreviations: ↑ (induce, regulating); ↓ (inhibit, lowering); ACE-I (angiotensin-converting enzyme Inhibitors); BP (blood pressure); DTHR (delayed-type hypersensitivity response); ICAM (intercellular adhesion molecule); IFN (interferon); MCP-1 (monocyte chemoattractant protein-1); PAG-AH (platelet activating factor acetylhydrolase); ROS (reactive oxygen species); TDAR (T-cell-dependent antibody response); TNF (tumor necrosis factor); VCAM (vascular cell adhesion molecule). * ND: information is not provided by the original article; **: the concentration indicated by original article.

## 3. Mechanisms of Actions of Selected Marine Peptides in Combating Cancer

The current rate of cancer occurrences is expected to reach 3.05 million by 2040, with an estimated mortality rate of nearly 7 million [2]. Common cancer treatments include chemotherapy, radiation, and surgery. However, chemotherapy has numerous side effects and can affect multiple organs. Over-expression of membrane transporters can lead to the expulsion of anticancer medicines, reducing their efficacy [20,21]. Peptides, due to their small size and chemical composition, can pass across cell membranes without causing harmful effects. They have high affinity and specificity, and few interactions with other medications. However, their limited bioavailability and activity compared to established cancer treatments pose challenges [22]. For instance, a peptide VECYGPNRPQF from *Chlorella vulgaris* was found to be an antiproliferative agent, inhibiting proliferation in the human gastric cancer cell line AGS but not in other cell lines, suggesting unique anticancer efficacy for certain tumor therapies [23]. Anticancer peptides found in marine species regulate various cellular and molecular pathways, including apoptosis, tubulin-microtubule balance, DNA defense, cell cycle control, migration, invasion, metastasis inhibition, and angiogenesis inhibition [11,24,25,26,27,28].

### 3.1. Apoptosis

**Figure 3:**
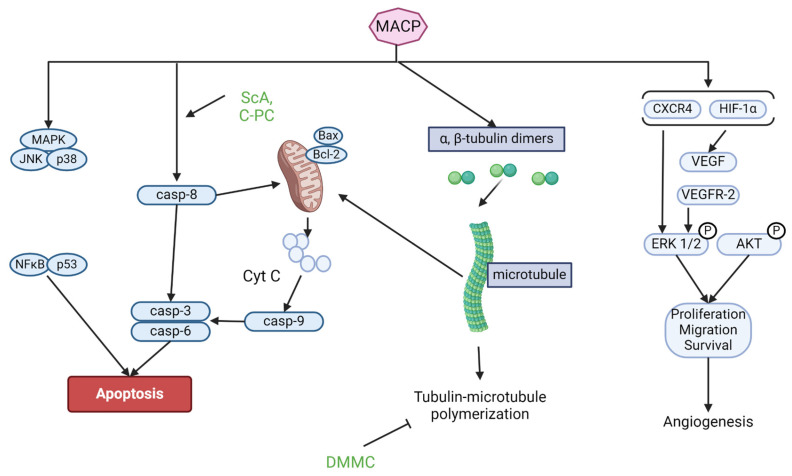
Anticancer effects of marine algal peptides. Abbreviations: Casp (Caspase); C-PC (C-phycocyanin); MACP (marine anticancer peptide); ScA (Somocystinamide A); VEGF (vascular endothelial growth factor).

Apoptosis is a critical process in development, physiology, and homeostasis. Its dysregulation, defined as the loss of pro-apoptotic signals or the gain of anti-apoptotic signals, can result in cancer genesis, development, and progression, as well as therapeutic failures. Apoptosis is a preferred method of cancer cell death during treatment because it does not normally elicit an inflammatory or immunological response. Pharmacological compounds that modulate apoptotic pathways and selectively induce apoptosis are potential approaches to cancer therapy [29,30,31,32,33]. Effective anticancer drugs should target many apoptotic pathways, both intrinsic and extrinsic. Caspase-3 activation occurs in intrinsic pathways, resulting in DNA damage, protein degradation, apoptosis, and cell uptake. Intrinsic routes, regulated by the Bcl-2 protein, produce Cyt C, whereas extrinsic pathways stimulate cell surface death receptors [34,35,36,37]. Some marine anticancer peptides activate the c-Jun N-terminal kinase (JNK) and MAPK pathways, causing cytochrome C (Cyt C) release from mitochondria, which initiates apoptosis by activating caspases and leading to cell death (Figure 3) [38]. Peptides such as Somocystinamide A and C-phycocyanin exhibit caspase-dependent anti-apoptotic activity in cancer cells [24].

### 3.2. Tubulin–Microtubule Balance

Marine anticancer peptide (MACP) kills cancer cells through mechanisms like disruption of the tubulin–microtubule balance [39]. Microtubules, formed from tubulin, are crucial for cell maintenance, transport, motility, and organelle distribution (Figure 3). Drugs that disrupt tubulin–microtubule equilibrium are effective cancer therapies [40]. The mitotic spindle, composed of microtubules and proteins, is crucial for cell division. Changes in the tubulin–microtubule balance can lead to cell degradation and death [41].

### 3.3. Angiogenesis

Angiogenesis, the development of new blood vessels, is vital in carcinogenesis, influencing solid tumor growth, invasion, and metastasis. It involves disrupting existing vessels, promoting endothelial cell proliferation, migration, and tube formation [42,43,44,45]. Vascular endothelial growth factor (VEGF) and its receptor, VEGFR-2, are critical in cancer angiogenesis (Figure 3). Cancer cells produce VEGF, stimulating angiogenesis via ERK1/2, CXCR4, HIF1α, and Akt. MMP2 and MMP9 are necessary for tumor invasion and metastasis. Blocking the VEGF-VEGFR-2 pathway and its downstream signals can slow tumor development. HIF1α controls adaptive responses to hypoxia and cellular functioning during normoxia, including VEGF aggregation. For instance, some peptides reduce MCF7 and MDA-MB-231 cell migration by reducing VEGFR2 expression and MMP-9 [46,47,48,49,50,51,52,53]. Mycothiazole from marine sponge, a mixed polyketide/peptide-derived molecule, suppressed hypoxia HIF1 signaling in tumor cells, decreasing HIF1 target gene VEGF production [54].

### 3.4. Cell Cycle Disturbance

Cell cycle disturbance is closely associated with apoptosis (Figure 3). Cyclin D1 and E inhibitors, p21 and p53, are activated to restrict tumor development and protect DNA from destruction by stopping the cell cycle and directing apoptosis [55,56,57,58,59,60,61,62]. For example, an undecapeptide derived from *C. vulgaris* protein waste with the sequence VECYGPNRPQF demonstrated significant dose-dependent antiproliferation and post-G1 cell cycle arrest in gastric cancer AGS cells with minimal cytotoxicity in normal lung fibroblast WI-38 cells [23]. Cyclodepsipeptides, including those derived from marine sponges, inhibit cell proliferation by disrupting microtubule dynamics and preventing proper mitotic spindle formation, which is crucial for cell division [63].

### 3.5. Membrane Disruption

MACP, as anticancer peptides depolarize cell membranes, cause tumor cells to lose osmotic pressure and spill cytoplasmic substances. They kill cancer cells using necrotic processes, resulting in membrane lysis and cell death. Peptides with low ROS activity may help avoid cancer [64,65,66,67,68,69,70].

## 4. Sensitization of Cancer Cells to Chemotherapy by Certain Algal

Cancer hallmarks refer to the common pathways that contribute to carcinogenesis, such as self-sufficiency, growth signaling, insensitivity to anti-growth signals, reproductive potential, tissue invasion, metastasis, resistance to apoptosis, sustained angiogenesis, immune surveillance evasion, tumor-promoting inflammation, genome instability, mutation, and cellular energetic dysregulation. These mechanisms can be effectively blocked by chemotherapies, yet its efficacy is eventually reduced following resistance growth after extended periods of exposure [71]. Drug resistance is a major concern in cancer treatment, and it is frequently caused by efflux, target alteration metabolism, cell surface receptor abnormalities, and epigenetic changes [72,73,74,75]. Therefore, sensitizing resistant cancer cells to the same or various medicines is of importance, allowing for the establishment of effective therapy regimens and overcoming a target shortage by using the same drug, but can facilitate the cancer cell death [76]. Recent anticancer medicines, such as small molecule targeted, immunotherapy, anti-angiogenic, peptide, protein, and gene therapies, have gained popularity because of their minimal side effects. Researchers find that algae show great promise to reduce cell proliferation, metastasis, and tumor angiogenesis while increasing apoptosis, indicating anticancer potential. Genetic modification also could improve their biological activity and enable focused cancer treatment [77].

The sensitization of cancer cells to chemotherapy by certain algae involves the use of algal-derived compounds to enhance the efficacy of chemotherapeutic agents. This process leverages the unique bioactive compounds found in algae, which can interact with cancer cells to increase their susceptibility to chemotherapy.

For example, phycocyanin from *Spirulina* has been shown to promote apoptosis in various cancer cells. C-phycocyanin, a new type of TAM-targeted photosensitizer, is efficient in in vitro photodynamic activity and selectively accumulates in tumor locations due to its affinity for tumor-associated macrophages (TAMs), providing a unique technique for improving cancer therapeutic efficacy [1,78]. It also contains peptides that have demonstrated potential in sensitizing cancer cells to chemotherapy by modulating pathways such as apoptosis, cell cycle arrest, and inhibition of drug efflux pumps [79]. Seaweed contains biologically active chemicals that induce death in cancer cells, making them more responsive to chemotherapy treatments [80]. Fucoidan, found in brown algae like *Fucus vesiculosus*, has demonstrated the ability to enhance the sensitivity of cancer cells to chemotherapy drugs like cisplatin and doxorubicin by inducing apoptosis and inhibiting cell proliferation [81,82].

## 5. Preclinical and Patents of Certain Algal Peptides as an Anticancer Agent

Several preclinical trials have reported the efficacy and safety of marine algal peptides in cancer therapy. These trials reported the potential of algal peptides to inhibit tumor growth, induce apoptosis, and enhance the effectiveness of conventional anticancer treatments. Several red and green algae species were included. Pal et. al. (2021) found that *Ulva intestinalis* and *Ulva lactuca* have the ability to reduce the proliferation of cervical cancer [83]. Two studies with the same cancer resulted in significant inhibition of the cell. Another study from Pradhan et. al. (2020) proved that *Enteromorpha compressa* increases apoptosis activity in oral cancer [84]. Furthermore, one study using a liver cancer cell line also worked as an anticancer by stimulating the marker of apoptosis [85]. Initial preclinical studies have shown promising results in different types of cancer, demonstrating the anticancer properties of certain algal peptides (Table 2).

**Table 2:** Preclinical Trial of Certain Algal Peptides as an Anticancer Agent.

References	Methods, Aim	Algae Species	Results
[83]	Methanolic extracts, Assess anticancer potential in cervical cancer cells (SiHa)	*Ulva intestinalis, Ulva lactuca*	- Algal fractions inhibited proliferation of SiHa cells in a dose-dependent manner - IC_50_ values against SiHa cells: 141.38 µg/mL (*U. intestinalis*) and 445.278 µg/mL (*U. lactuca*)
[84]	Methanolic extracts, Assess anticancer potential in oral squamous cell carcinoma (OSCC)	*Enteromorpha compressa*	- Methanolic extract of *E. compressa* exhibited robust free radical scavenging activity - Enhanced intrinsic apoptosis against OSCC by downregulating protective antioxidant enzymes - Induction of autophagy to promote cell death in oral cancer cells
[85]	Aqueous extracts, Assess antiviral potential in HeLa cells co-cultured with HTLV-I infected-T-cell line (causative agent of adult T-cell leukemia/lymphoma)	*Ulva fasciata, Sargassum vulgare, Vidalia obtusiloba, Laminaria abyssalis*	- *U. fasciata* extract showed 60.2% syncytium inhibition at 2.5% concentration - *S. vulgare* and *V. obtusiloba* extracts presented 78.8% and 76% syncytium inhibition, respectively, at 5% concentration - *L. abyssalis* extract exhibited 100% syncytium inhibition at 2.5% concentration
[86]	Methanolic extracts, Assess anticancer potential in HeLa cells	*Enteromorpha intestinalis*, *Rhizoclonium riparium*	- IC_50_ values of 309.048 ± 3.083 μg/mL (E. intestinalis) and 506.081 ± 3.714 μg/mL (R. riparium) - Treated cells exhibited morphological changes including rounding with blebbing and condensed nuclei - Formation of acidic lysosomal vacuoles observed in treated cells - Expression of apoptotic genes in both mRNA and protein levels decreased - Expression of LC3B-II suggested occurrence of autophagy in treated cells
[87]	Assess anticancer potential in Human lung cancer cell lines (A549, H460 and H1299) and lung fibroblast MRC-5 cells	*Bryopsis plumosa*	- Treated cells exhibited morphological changes involved in the typical EMT and apoptosis - Expression of E-cadherin increased - Expression of N-cadherin, Zeb1, snail and vimentin decreased - Suppressed migration and invasion in NSCLCs
[88]	Assess anticancer potential in HT29 and LS174 cells	*Pterocladiella capillacea*	- Decreased the viability of LS174 and HT29 cells in a dose-dependent manner - IC50 values of 56.50 ± 8.68 µg/mL (HT29 cells) and 49.77 ± 4.51 µg/mL (LS174 cells) - Enhanced of AKT and ERK-1/-2 activation
[89]	Assess potential anticancer in MDA-MB-231, MDA-MB-453, MCF7, A549, H1299, HCT116, SW620, CT26, PC3, DU145, HeLa	*Sargassum macrocarpum*	- Induced apoptosis -Expression of Bcl2 decreased - Expression of cleaved caspase-3 and PARP increased - Enhanced DNA fragmentation - STAT3 signaling pathway inhibition

Marine algae peptides constitute a burgeoning class of therapeutic agents of cancer treatment [83,84]. However, their clinical utility is often curtailed by challenges pertaining to bioavailability and susceptibility to enzymatic degradation within biological systems [90,91]. Marine algae peptides have low capacity to attain therapeutic concentrations at target sites [92]. These hurdles underscore the critical need for innovative approaches to unlock the full therapeutic potential of marine algal peptides.

Several patents have reported the use of algal peptides as anticancer agents, indicating commercial interest and the potential for future therapeutic applications. These patents cover the identification of novel peptide sequences, methods for peptide synthesis, and formulations for enhancing peptide stability and bioavailability (Table 3). Moreover, patents may also address the use of algal peptides in combination with therapies or targeted drug delivery systems for improved cancer treatment outcomes by using nanotechnology.

The use of nanocarrier-based delivery systems as a strategy for augmenting the bioavailability of marine algal peptides has been increasing currently [93]. Nanocarriers, encompassing liposomes, nanoparticles, and micelles, offer distinctive advantages in modulating the pharmacokinetic profiles and tissue distribution of peptides. The encapsulation of marine algal peptides within nanocarriers affords protection against enzymatic degradation and facilitates controlled release kinetics, thereby enabling sustained drug delivery and optimized therapeutic efficacy [92].

Preliminary investigations into nanocarrier-based delivery systems have yielded encouraging findings in preclinical models [94]. Liposomal formulations have demonstrated proficient encapsulation of marine algal peptides, yielding improvements in solubility, stability, and in vivo bioavailability [95]. Similarly, nanoparticle-based delivery platforms have exhibited enhanced pharmacokinetic profiles and augmented tissue distribution of marine algal peptides, thereby heralding enhanced therapeutic efficacy [96].

The translation of these preclinical endeavors into clinical practice holds profound implications for cancer therapy [97]. By circumventing the obstacles associated with the bioavailability of marine algal peptides, nanocarrier-based delivery systems offer a transformative pathway toward more efficacious and targeted anticancer interventions [98]. Moreover, their potential for synergistic combination therapies and tailored therapeutic regimens underscores their pivotal role in cancer treatment paradigms [99].

**Table 3:** Patents of certain algal peptides as an anticancer agent.

Inventor, Year	Country	Identifier	Polypeptide Names	Method	Type of Formulations
Figueirdo et al., 2018 [100]	China	CN104812381B	No specific data	Therapeutic nanoparticle preparation	Nanoparticles for targeted drug delivery
Miller et al., 2017 [101]	USA	US9668951B2	No specific data	Pharmaceutical compositions comprising renewably based biodegradable 1,3-propanediol	Oral, topical, or injectable formulations include biodegradable pharmaceutical compositions
Lin et al., 2014 [102]	USA	US8859727B2	Fused in sarcoma-1	Nanoparticle–polypeptide complexes	Bioactive peptide–nanoparticle complexes
Aharoni et al., 2024 [103]	USA	US20200354759A1	Cyp76ad1-beta clade	Genetic engineering	Polynucleotide-encoded polypeptides
Foger et al., 2021 [104]	USA	US10905744B2	Glucagon-like peptide-1	Oral delivery drugs	Peptide drugs formulations
Bradbury et al., 2023 [105]	Canada	CA2900363C	Tyr3-octreotide	Silica-based nanoparticles	Multimodal silica-based nanoparticle formulations
Klein et al., 2019 [106]	USA	US20190022228A1	Glucagon-like peptide-1	Microparticle/nanoparticle formulations	Drug delivery particles

## 6. Current Challenges and Future Perspectives for Using Peptides as Anticancer Agents

Ensuring their stability and bioavailability poses a significant challenge. Their susceptibility to enzyme degradation and their characteristics of poor absorption and rapid clearance are challenges in their application as a therapeutic agent for anticancer [90]. Moreover, large-scale production, while maintaining the quality and activity of peptides, is technically demanding and expensive [107]. Lastly, a lack of clinical studies evaluating the safety, efficacy, and pharmacokinetics of marine algal peptides in cancer therapy presents challenges on the implementation of this novel strategy in clinical settings.

Future research should prioritize enhancing the stability and bioavailability of marine algal peptides. The advanced exploration of marine algal biodiversity may aid the discovery of novel peptides with potent anticancer properties. The use of marine algal peptides as multimodal cancer treatment regimens should be studied. Finally, collaborative efforts between researchers, industries, and regulatory agencies are needed to advance promising peptide candidates from the laboratory to clinical application.

## 7. Conclusion and Highlights

In conclusion, marine algal peptides hold a promising role in cancer therapy. Investigating the anticancer properties of marine algal peptides is crucial for improving clinical modalities, particularly in the development of anticancer drugs with minimum adverse effects. Peptides are believed to be non-harmful because they penetrate cellular membranes through a specific mechanism attributed to their small size and unique chemical properties. Marine algae have been shown to act as rich sources of bioactive compounds, including peptides, with potent anticancer properties. Among the bioactive compounds identified, specific peptides from the cyanobacterium *Lyngbya majuscula*, such as Isomalyngamide A and A-1, have demonstrated particularly potent anticancer properties by inhibiting VEGFR2 and MMP-9, which are critical factors in tumor growth and metastasis. Moreover, peptides such as VECYGPNRPQF from *Chlorella vulgaris* have exhibited significant antiproliferative effects, particularly against the gastric cancer cell line AGS, underscoring their potential as promising candidates for further development in cancer therapy. Notably, this peptide also demonstrated minimal cytotoxicity to the lung fibroblast WI-38 cells, highlighting its therapeutic specificity and safety profile.

Mechanisms underlying the anticancer activities by marine algal peptides are varied, including apoptosis induction, tubulin-microtubule balance disruption, angiogenesis inhibition, cell cycle disturbance, and membrane disruption. Somocystinamide A and C-phycocyanin are examples of algal peptides that have been reported to induce cancer cell apoptosis through the caspase pathway. Apoptosis by marine algal peptides may also involve the release of Cyt C concomitant to the activation of the JNK and MAPK pathways.

Despite the potential, the efficacy of the algal peptide could be challenged by the complex physiological response which contributed to low bioavailability and bioaccessibility. Therefore, innovative approaches, such as nanocarrier-based delivery systems, have been proposed to overcome challenges associated with the bioavailability and stability of marine algal peptides. Nanocarriers, including liposomes, nanoparticles, and micelles, enhance the pharmacokinetic profiles and tissue distribution of these peptides. Encapsulation within nanocarriers protects the peptides from enzymatic degradation and enables controlled release, thereby improving therapeutic efficacy.

Continued exploration and clinical trials are essential to validate their efficacy and safety, optimize delivery systems, and develop targeted therapeutic regimens. The sensitizing activity of the peptide against cancer cells, which can improve the efficacy of chemotherapy drugs (such as cisplatin and doxorubicin), is also an interesting research topic that is worth further exploration. The integration of marine algal peptides into cancer treatment paradigms could offer more effective and targeted interventions, ultimately advancing the fight against cancer.

## Data Availability

All data analyzed during this study are included in this published article.
